# Historical trends in modifiable indicators of cardiovascular health and self-rated health among older adults: Cohort differences over 20 years between the Berlin Aging Study (BASE) and the Berlin Aging Study II (BASE-II)

**DOI:** 10.1371/journal.pone.0191699

**Published:** 2018-01-31

**Authors:** Maximilian König, Johanna Drewelies, Kristina Norman, Dominik Spira, Nikolaus Buchmann, Gizem Hülür, Peter Eibich, Gert G. Wagner, Ulman Lindenberger, Elisabeth Steinhagen-Thiessen, Denis Gerstorf, Ilja Demuth

**Affiliations:** 1 Lipid Clinic at the Interdisciplinary Metabolism Center, Charité – Universitätsmedizin Berlin, Berlin, Germany; 2 Department of Psychology, Humboldt University Berlin, Berlin, Germany; 3 Geriatrics Research Group, Charité – Universitätsmedizin Berlin, Berlin, Germany; 4 Department of Nutrition and Gerontology, German Institute of Human Nutrition, Potsdam-Rehbrücke, Germany; 5 Department of Psychology, University of Zurich, Zurich, Switzerland; 6 University Research Priority Program (URPP) Dynamics of Healthy Aging, University of Zurich, Zurich, Switzerland; 7 Health Economics Research Centre, University of Oxford, Oxford, United Kingdom; 8 German Institute for Economic Research, Berlin, Germany; 9 Max Planck Institute for Human Development, Berlin, Germany; 10 Center for Lifespan Psychology, Max Planck Institute for Human Development, Berlin, Germany; University College London, UNITED KINGDOM

## Abstract

**Background:**

The last decades have seen great advances in the understanding, treatment, and prevention of cardiovascular disease (CVD). Although mortality rates due to CVD have declined significantly in the last decades, the burden of CVD is still high, particularly in older adults. This raises the question whether contemporary populations of older adults are experiencing better or worse objective as well as subjective health than earlier-born cohorts. The aim of this study was to examine differences in modifiable indicators of cardiovascular health (CVH), comparing data obtained 20 years apart in the Berlin Aging Study (BASE, 1990–93) and the Berlin Aging Study II (BASE-II, 2009–2014).

**Methods:**

Serial cross-sectional analysis of 242 propensity-score-matched participants of BASE (born 1907–1922) and BASE-II (born 1925–1942). Body mass index (BMI), blood pressure, total cholesterol, glycated hemoglobin (HbA1c), diet, smoking and physical activity were operationalized according to the “Life’s simple 7“(LS7) criteria of the American Heart Association.

**Results:**

121 matched pairs were identified based on age, sex, and education. In the later-born BASE-II sample, the mean LS7 score was significantly higher than in the earlier-born sample (7.8±1.8 vs. 6.4±2.1, p<0.001), indicating better CVH. In detail, diet, physical activity, smoking, cholesterol, and HbA1c were more favorable, whereas blood pressure was significantly higher in individuals from the later-born cohort. BMI did not differ significantly between the two matched samples. Notably, despite better CVH, later-born individuals (BASE-II) reported lower self-rated health, presumably because of higher health expectations.

**Conclusions:**

Overall, cardiovascular health was significantly better in the later-born cohort, but several notable exceptions exist.

## Introduction

Mortality rates due to cardiovascular disease (CVD) have decreased significantly among all age groups during the past decades in high-income countries, which is attributed to reductions in risk factors and improvements in treatments [1, 2]. Still, CVD imposes a huge burden in terms of mortality, morbidity, disability, functional decline, and healthcare costs, particularly in people aged 60 years and older [3].

The traditional cardiovascular risk factors adiposity, blood pressure, cholesterol, insulin resistance and diabetes, and smoking, as well as physical inactivity operate into old age. Thus, primary, secondary and tertiary prevention among older people hold huge potential [4]. Over the past 20 years, there have been considerable advances in cardiovascular medicine in terms of understanding, diagnosis, therapy, and prevention. One prominent example is the use of statins (HMG-CoA reductase inhibitors) for secondary prevention: The first of class was launched 1987. Their value for risk reduction was only evidenced in 1994 by the 4S-study and their use has been ever-increasing since [5]. Likewise, diabetes and hypertension have seen important advances. For example, the fasting glucose cut-off levels for diagnosing diabetes were lowered from 140 mg/dl to 126 mg/dl in 1997, after important landmark trials had demonstrated the benefits of tight glycemic control [6], and the blood pressure target of <140/90 mmHg was established only in 1998 [7], just as numerous new drugs were (re-)launched in this period, e.g. metformin.

Furthermore, behavioral risk factors were also subject to desired and undesired historical trends. First, the use of tobacco decreased in the past 20 years, among other things because smoke-free laws have been established since the middle of the first decade of the 21^st^ century [8]. In a similar vein, evidence of the benefits of what is referred to as Mediterranean diet (replacing saturated fats with unsaturated fats, low dietary salt intake, and diets rich in fruits, vegetables, whole grains, and nuts) has accumulated over the last decades and has been translated into guidelines [9, 10]. In contrast, universal secular trends of ever-rising BMI and increasing prevalence of type 2 diabetes in the last decades are rather disillusioning [11, 12].

Against this background of major advances but also setbacks, the question arises whether contemporary populations of older adults have a more favorable profile of cardiovascular health (CVH) indicators relative to their earlier-born peers. In the present study, we aimed to describe cohort differences in CVH indicators between two cohorts of community-dwelling older adults. We used serial cross-sectional data from the two successive Berlin Aging Studies (BASE and BASE-II), whose participants were born 20 years apart in 1907–1922 (BASE) and in 1925–1942 (BASE-II).

In order to operationalize CVH, we made use of the Life's Simple 7 (LS7) metrics [13], a concept proposed by the American Heart Association (AHA). Meeting the ideal levels for seven health behaviors and factors, including smoking, physical activity, body mass index, diet, total cholesterol, blood pressure, and blood glucose, is considered as “ideal cardiovascular health”.

Additionally, we contrasted the observed historical changes in cardiovascular health factors and behavior with historical trends in self-rated health (SRH).

## Materials and methods

We used baseline data from the Berlin Aging Study (BASE; obtained in 1990–1993) and the Berlin Aging Study II (BASE-II; obtained in 2009–2014). Detailed descriptions of participants, variables, and procedures can be found in previous publications [14]. Details, which are relevant in the context of the current analysis, are presented below.

### Participants and procedure

#### BASE

The initial BASE cohort consisted of 516 residents of former West-Berlin districts (age: *mean* = 84.9, *SD* = 8.7, range = 70–103 years; 50% women), identified based on the obligatory city registry [15]. 443 of the initial 516 BASE participants were eligible for inclusion in the propensity-score matching procedure. Finally, we used data from 121 participants (age: mean = 74.3 years, SD = 3.0, range = 70–84 years; 48.8% women). Participants in the matched BASE sample were born 1907 through 1922 (Median = 1917; SD = 3.6 years).

#### BASE-II

The BASE-II cohort was drawn as a convenience sample from the greater Berlin metropolitan area. 2,172 participants (~ 75% aged 60–84 years and ~25% aged 20–35 years) were recruited for the medical part of the study [14, 16]. For our current analysis, all BASE-II participants >60 years with data on the relevant study variables available (n = 1293) were eligible for inclusion in propensity-score matching procedure. Finally, we used data from 121 participants (age: mean = 74.3 years, SD = 3.1, range = 70–84 years; 51.2% women). Participants in the matched BASE-II sample were born 1925 through 1942 (Median = 1938; SD = 3.3 years).

All participants gave written informed consent. The Ethics Commission of the Berlin Chamber of Physicians (Ärztekammer Berlin) approved the BASE study prior to the first assessments in 1990 (the approval was not numbered), and the Ethics Committee of the Charité-Universitätsmedizin Berlin approved the BASE-II study (approval number EA2/029/09).

### Propensity score matching

To minimize possible confounding and equate the cohort samples as closely as possible with respect to socio-demographic factors (age, gender, cohort-normed education), we used propensity score matching procedures [17].

**Fig 1:**
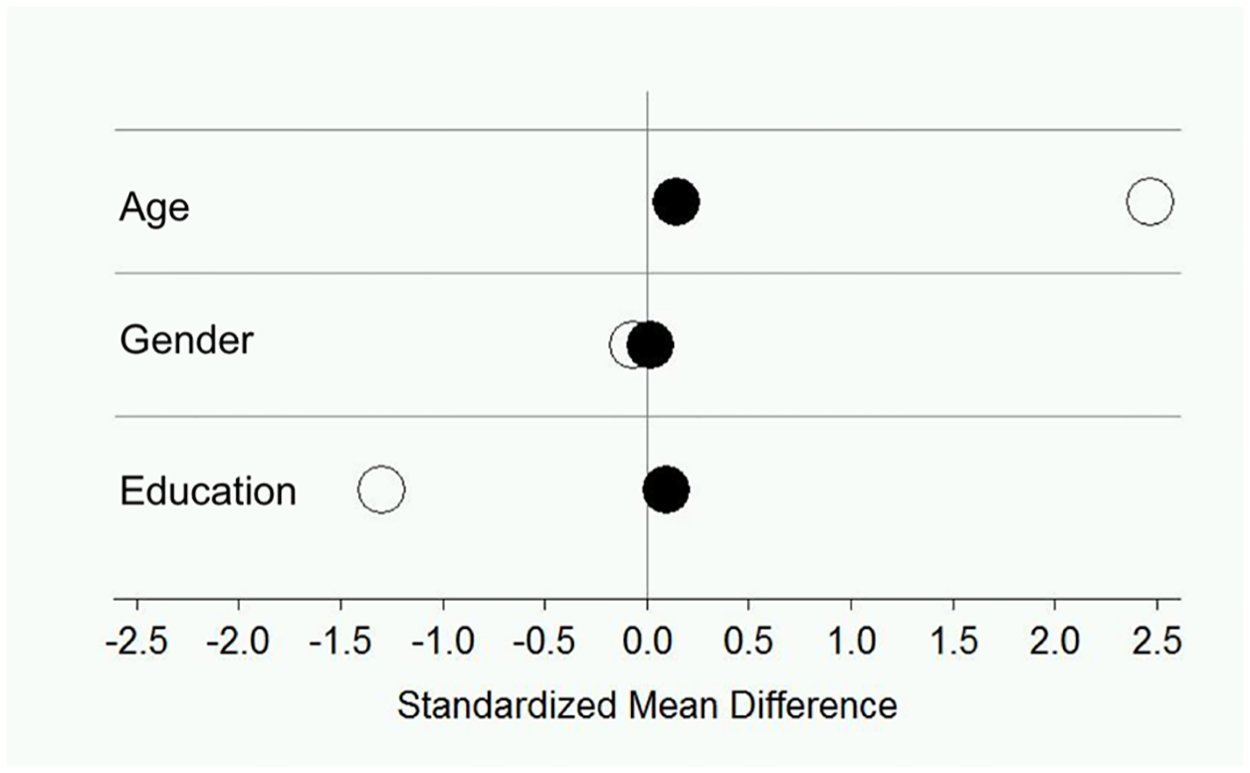
BASE and BASE-II before and after propensity score matching. Illustrating standardized mean differences between the Berlin Aging Study (BASE) and Berlin Aging Study-II (BASE-II) cohorts/samples in sociodemographic variables. Negative (positive) numbers signify greater scores for BASE (BASE-II) participants (unfilled circles). After the matching (black circles), the differences were small and not reliably different from 0 at p < 0.05.

Age was calculated as the difference between the date of the interview and a participant’s date of birth and scaled in years. Cohort-normed education was measured as the number of years the individual had spent in formal schooling and standardized by cohort using data of the appropriate reference groups (e.g., for BASE: mean = 10.90 years, SD = 1.99, and for BASE-II: mean = 14.10 years, SD = 2.86). Calculating a logistic regression, we used 1:1 matching methods to select for each participant from the BASE cohort (*n* = 443) a “twin” participant from the BASE-II cohort (*n* = 1293), who was the same or as similar as possible on the matching variables. To calculate a between-groups distance matrix, the propensity score was logit-transformed as recommended in the propensity score matching literature (e.g. Rosenbaum & Rubin, 1985 [18]). We matched nearest neighbors with a caliper-matching algorithm. The caliper (maximum allowable distance between matched participants) was continuously increased by steps of 0.001 until cohort differences in the matching variables were no longer reliably different from 0 at *p* < 0.05. Each participant in BASE was allocated the nearest neighbor from BASE-II only if the neighbor fell within the caliper distance. With a caliper of c < 0.9 *SD*, the matched BASE-II and BASE samples no longer differed in age, gender, and cohort-normed education. A suitable neighbor in BASE-II could be identified for 121 BASE participants. Fig 1 shows standardized mean differences between the cohorts/matched samples on the matching variables before and after the propensity score-matching. Descriptive statistics for study measures are given in Table 1 separately for the matched samples.

**Table 1:** Characteristics of propensity score matched samples.

	BASE N = 121	BASE-II N = 121	*p*
**Socio-demographic characteristics**			
Age, years	74,3±3,0	74,3±3,0	0.891
Sex, female (%)	48.8	51.2	0.700
Cohort-normed education	-0.2(-0.5,0.4)	-0.2(-0.9,1.4)	0.244
**Physical health characteristics**			
Systolic blood pressure, mmHg	143.4±21.7	149.8±21.3	0.022
Diastolic blood pressure, mmHg	80.6±12.9	83.7±11.6	0.049
Heart rate, bpm	69.9±12.5	70.4±10.7	0.720
Body-mass-index, kg/m^2^	26.7±3.9	27.2±4.5	0.322
Waist circumference, cm	93.8±11.1	98.4±12.7	0.003
HbA1c,%	5.9(5.5,6.7)	5.7(5.4,5.9)	0.002
Total cholesterol (mg/dl)	243.3±41.5	219.6±36.7	<0.001
LDL-Cholesterol (mg/dl)	156.8±37.4	135.3±34.1	0.001
HDL-Cholesterol (mg/dl)	55.2±15.8	63.7±14.6	<0.001
Lipid-lowering medication (%)	9.1	17.4	0.103
Antidiabetic drugs (%)	9.1	8.3	0.450
Antihypertensive drugs (%)	51.2	62.0	0.519
Self-reported diabetes (%)	10.7	10.7	1.000
Self-reported hypertension (%)	27.3	58.7	<0.001
Self-reported hyperlipidemia (%)	10.7	34.7	<0.001
Cardiovascular disease (%)	25.6	15.4	0.051

Abbreviations: BASE, Berlin Aging Study; BASE-II, Berlin Aging Study II; LDL, low density lipoprotein; HDL, high density lipoprotein; HbA1c, glycated hemoglobin. Data are given as mean ± standard deviation, median (interquartile range) or percentages. Two-sample t-test was used for comparison of means and Mann-Whitney-U test for comparison of medians. Proportions were compared with the χ^2^-test.

### Measurement and definition of Life’s simple 7 (LS 7) metrics

We measured Life’s Simple 7 health behaviors and health factors according to definitions published by the AHA [13]. We adapted definitions for healthy diet and physical activity components based on data available in BASE and BASE-II, as described below and in Table 2. Following established procedures, LS7 items (smoking, body mass index (BMI), physical activity, diet, blood pressure (BP), total cholesterol (TC), and HbA1c) were categorized into ideal (2 points), intermediate (1 point), and poor (0 points), adding up to a total score ranging from 0 to 14 points (see Table 2). As per convention, CVH of study participants was graded as *inadequate (0 to 4 points)*, *average* (5 to 9 points) and *optimal* (10 to 14 points) (Table 2).

**Table 2:** Distribution of Life’s simple 7 (LS7) metrics in the matched BASE and BASE-II samples.

	Score	BASE (n = 121)	BASE-II (n = 121)	*p*
**Blood pressure, mmHg**				
<120/80, unmedicated	2	8.3%	2.5%	0.108
120 to 139/80 to 89 or treated to goal	1	28.3%	26.4%	
≥140/90	0	63.3%	71.1%	
**Total serum cholesterol, mg/dL**				
<200 mg/dL, unmedicated	2	12.6%	21.0%	0.001
200–239 mg/dL or treated to <200 mg/dL	1	37.8%	52.1%	
≥240 mg/dL	0	49.6%	26.9%	
**Hemoglobin A1C**, %				
<5.7%, unmedicated	2	35.5%	46.2%	0.001
5.7 to 6.4 or treated to <5.7%	1	33.9%	45.4%	
>6.4%	0	30.6%	8.4%	
**Smoking**				
Never or quit >12 months ago	2	76.9%	95.9%	< 0.001
Former ≤12 months	1	3.3%	0.0%	
Current	0	19.8%	4.1%	
**Body mass index, kg/m**^**2**^				
<25	2	34.2%	33.3%	0.325
25 to 29.9	1	49.2%	42.5%	
≥30	0	16.7%	24.2%	
**Physical activity**				
Several times a week	2	40.5%	50.0%	0.002
At least once a week	1	11.6%	23.2%	
Infrequently/never	0	47.9%	26.8%	
**Healthy diet**				
Healthy diet score 5-6/6	2	9.1%	28.9%	< 0.001
Healthy diet score 3-4/6	1	48.8%	50.4%	
Healthy diet score 0-2/6	0	42.1%	20.7%	
**CVH**				
Optimal	10–14	6.6%	17.4%	<0.001
Average	5–9	73.6%	79.3%	
Inadequate	0–4	19.8%	3.3%	
Mean LS7 Score		6.4±2.1	7.8±1.8	<0.001

Notes: BASE = Berlin Aging Study, CVH = cardiovascular health. Data are given as mean ± standard deviation or percentages. Χ^2^-test was used to assess differences in distribution of categorical variables and two-sample t-test was used for comparison of the mean LS7 score.

*Smoking* status was assessed as part of the comprehensive medical history, taken by physicians. Participants were classified as current smokers (0 points), former smokers, if they quit within the past 12 months (1 point), or never smokers, if they had never smoked or quit more than 12 months ago (2 points).

*BMI* was calculated from objective height and weight measurements, using the standard formula, weight in kilograms divided by height in meters squared.

*Physical activity* was measured using the Baecke physical activity questionnaire (BASE II) [19] and congruent questions from the BASE-questionnaire. The frequency of exercise was categorized into never/seldom (0 points), sometimes (1 point), often/always (2 points).

*Diet* was assessed using self-report questions from the BASE-questionnaire. Participants were asked: “How often do you eat …?”, and the possible answers were (almost) every day, several times per week, once a week, 2 to 3 times per month, once a month or less, never. For BASE-II, the far more detailed and comprehensive EPIC-food frequency questionnaire [20] was available. The final components of our healthy diet score were:

1. fruits ≥ 1 servings/day
2. vegetables ≥ 1 servings/day
3. fish ≥ 2 servings/week
4. sugar-sweetened beverages ≤ 1 servings/week
5. processed meats ≤ 1.5 servings/week
6. unprocessed red meats ≤ 1.5 servings/week

A healthy diet score of 5–6 was considered ideal (2 points), 3–4 was considered intermediate (1 point), and 0–2 was classified as poor (0 points).

*Blood pressure* (BP) measurements were taken in a seated position after participants had rested for 5 minutes. The mean of two measurements (right and left arm) was used for analysis.

*Total cholesterol*, *LDL-cholesterol*, *glycated hemoglobin (HbA1c) and glucose* were measured centrally in a certified laboratory. As we could not ascertain that blood glucose levels had been obtained from fasting blood samples in BASE, we chose to use HbA1c for the categorization into the LS7 metrics.

Both in BASE and BASE-II, participants were asked to bring the medication packets for all drugs used on a regular basis, as well as their medication plan. Study staff reviewed the medication and took a comprehensive medication history (incl. indication, dosage, start, and side-effects). Self-reported diagnoses (i.e., diabetes, hypertension, dyslipidemia) were obtained during medical history taking.

### Self-rated health (SRH)

Self-rated health was assessed in BASE and BASE-II with a single question: “How do you rate your current health?” with an answering scale ranging from 1 (“very good”) to 5 (“poor”).

### Statistics

Results are presented as mean ± standard deviation (SD), as median with interquartile range (IQR), or as percentages. Two-sample t-test was used for between-group comparison of continuous variables with a normal distribution, and Mann–Whitney U test was used for comparison of skewed continuous or discrete variables. The normality assumption was assessed by visual inspection of the distribution in conjunction with the Shapiro-Wilk test. Proportions were compared with the Chi^2^ test. Statistical significance was evaluated at p <0.05. We used linear regression models to adjust for minor disparities in the prevalence of cardiovascular outcomes (history of stroke and/or coronary heart disease), and to examine the role of cohort membership, age, sex, and education for the LS7 count. The combined variable “CV disease” was imputed as a binary variable. We also tested quadratic (e.g., for chronological age) and interaction effects with the cohort variable and retained in the final models only those that had emerged as statistically significant.

We used IBM SPSS Statistics version 23 and SAS 9.2. Power analyses were performed using the G*Power software [21].

## Results

### Cohort differences in LS7 score

In the matched samples, mean age was age 74.3 years±3.0 years (BASE) and 74.3±3.1 years (BASE-II) and 48.8% and 51.2% were female, respectively (Table 1).

**Fig 2:**
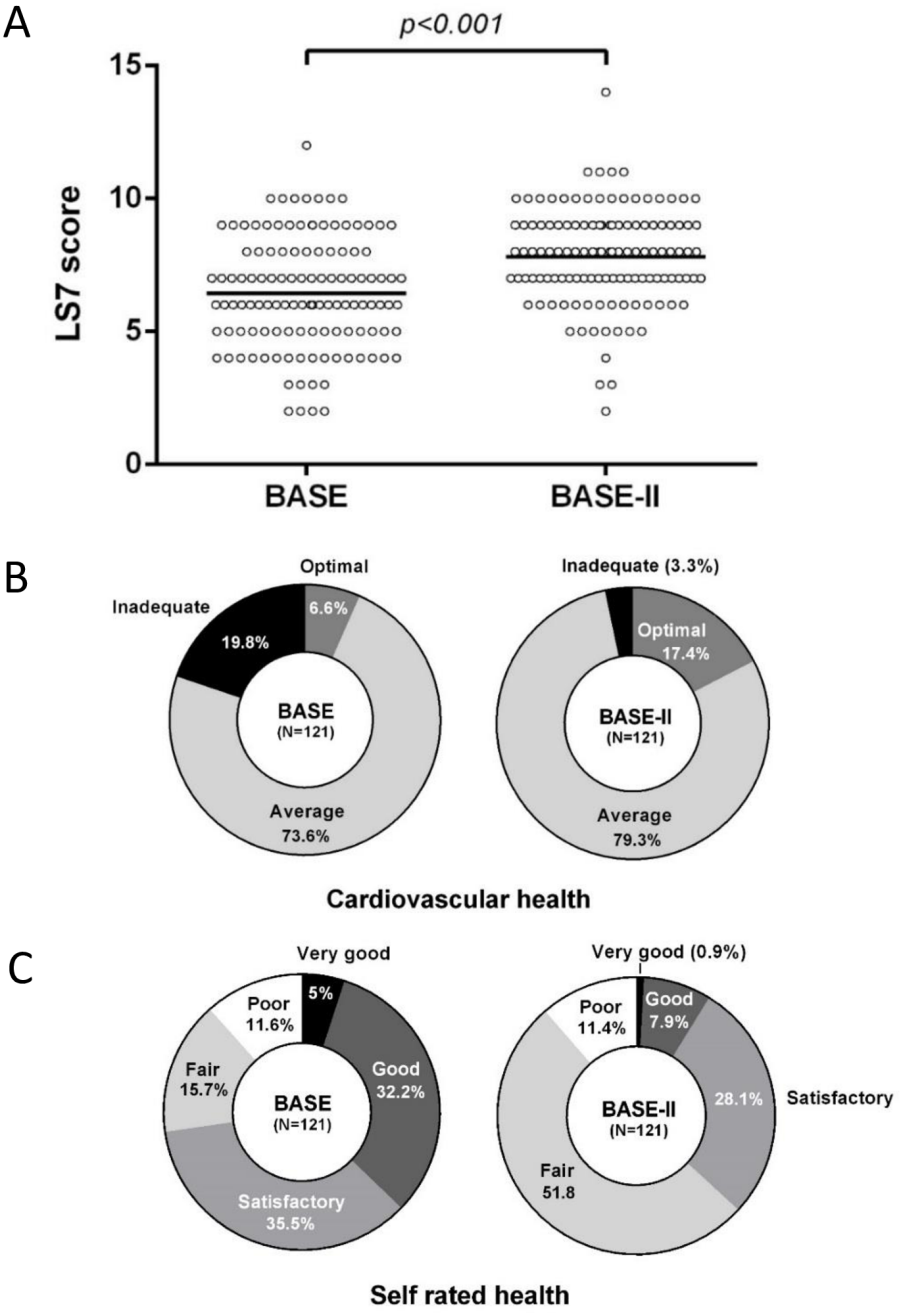
Cardiovascular and self-rated health in BASE and BASE-II. **A)** Aligned dot plots showing the distribution of the Life’s Simple 7 (LS7) score in the matched samples of BASE (n = 121) and BASE-II (n = 121). The LS7 score ranges from 0–14, and a higher score indicates better cardiovascular health. The mean, indicated by the horizontal line in the center, was 6.4±2.1 in BASE and 7.8±1.8 in BASE-II (p <0.001). **B)** Proportions of matched BASE and BASE-II participants meeting the criteria for inadequate, average or optimum cardiovascular health (CVH), according to the concept of the Life’s Simple 7. **C)** Self-rated health of matched BASE and BASE-II participants: Proportions of very good (black), good (dark grey), satisfactory (medium grey), fair (light grey), poor (white).

Overall, the mean LS7 score was significantly higher in the BASE-II sample than in the earlier-born BASE participants (7.8±1.8 vs. 6.4±2.1, p<0.001), indicating better CVH (Fig 2A). In both matched samples, from BASE and BASE-II, distribution of LS7 scores was approximately normal. As illustrated in Fig 2A, only one participant out of 121 in the BASE-II sample, and no one in BASE met all seven criteria for ideal CVH (LS7 score = 14).

According to the LS7 metrics, the majority of the 242 matched BASE and BASE-II individuals had average cardiovascular health (73.6% and 79.3%, respectively). Whereas only a small proportion met the criteria for “optimal CVH” in BASE (6.6%), 17.4% of the BASE-II sample had “optimal CVH” (Table 2). Furthermore, only few BASE-II participants (3.3%), but one fifth of all BASE-participants (19.8%) had “inadequate CVH” (Table 2, Fig 2B).

### Cohort differences in diet, physical activity, and smoking

A remarkable difference was evident in subjects’ dietary habits. 28.9% of the later-born subjects (BASE-II) showed an ideal healthy diet, fulfilling 5–6 of maximum six healthy diet criteria, compared to only 9.1% in the matched BASE sample. Conversely, in 42.1% vs. only 20.7% (BASE vs. BASE-II) dietary habits were poor (0–2 of 6 criteria).

A concordant trend was observed in the extent to which subjects reported being physically active. Whereas among the earlier-born subjects, almost 50% reported to do no or virtually no exercise, in the later-born sample only 26.8% were physically inactive. Likewise, the proportion of subjects who reported to be physically active on a very regular basis was significantly larger in BASE-II (50% in BASE-II vs. 40% in BASE). Furthermore, the percentage of current smoking was also significantly lower in the BASE-II (4.1%) than in the BASE sample (19.8%), with similar proportions among men (3.4% vs. 22.6%) and women (4.8% vs. 16.9%) in BASE and BASE-II, respectively.

### Cohort differences in body mass index

There was no significant difference regarding proportions of ideal, intermediate, and poor BMI comparing earlier-born and later-born individuals (p = 0.150). Notably, approximately 70% of individuals were overweight or obese in both samples, respectively. The mean BMI was 27.2 kg/m^2^ (BASE-II) and 26.7 kg/m^2^ (BASE), respectively (p = 0.322). A post-hoc power analysis indicated that we might have missed an existing difference due to small sample size: e.g. a sample of 479 participants would have been required in order to detect a difference (80% chance) in proportions of obese subjects (BMI≥30) at the 5% level. We therefore also regarded waist circumference as another obesity measure. The mean waist circumference was indeed significantly higher in the later-born sample (98.4 cm vs. 93.8 cm, p = 0.003), which was mainly due to a pronounced difference in men (102.6 cm in BASE-II vs. 96.7 cm in BASE, p = 0.004).

### Cohort differences in blood pressure

According to the LS7 metrics, large proportions of both matched samples, BASE (63.3%) and BASE-II (71.1%), were hypertensive (BP ≥140/90 mmHg), whereas only small proportions (8.3% and 2.5%, respectively) showed ideal, unmedicated BP <120/80 mmHg (p = 0.108). When considering BP as non-categorized variables, both mean systolic blood pressure (SBP) and diastolic blood pressure (DBP) were significantly higher in BASE-II than in BASE (Table 1). This trend was found statistically significant only for SBP in men (men 149/83 vs. 140/80 mmHg, p = 0.015/0.110; women 150/83 vs. 146/81 mmHg, p = 0.376/0.245).

The frequency of BP medication use was similar in BASE-II and BASE (62% and 51.2%, respectively), however, the preferred drugs had changed over time (BASE: nifedipine, thiazides, atenolol, reserpine, triamterene; BASE-II: hydrochlorothiazide, ramipril, metoprolol, amlodipine, valsartan). Interestingly, the prevalence of self-reported hypertension was nearly twice as high in the BASE-II sample, as in BASE (58.7% vs. 27.3%, p <0.001).

### Cohort differences in total cholesterol

In terms of LS7 metrics, 21% of the matched BASE-II sample showed ideal untreated total cholesterol (TC) levels, compared to only 12.6% in BASE. Notably, half (49.6%) of the earlier-born BASE sample had poor TC levels (i.e. ≥240 mg/dl), compared to only 26.9% in BASE-II (p = 0.001). The mean TC was significantly lower in the later-born individuals than in the earlier-born individuals (220 vs. 243 mg/dl, p<0.001). This difference was more pronounced among men (Δ = 35 mg/dl, p<0.001) than women (Δ = 15 mg/dl, p = 0.002). Both the prevalence of lipid-lowering drug use and the prevalence of self-reported hyperlipidemia were significantly higher in the later-born sample.

In more detail, the mean LDL-cholesterol was significantly lower, while HDL-C was significantly higher in BASE-II than in BASE (Table 1).

### Cohort differences in glycated hemoglobin (HbA1c)

In the matched BASE-II sample, 46.2% showed ideal HbA1c, compared to only 35.5% in BASE. Furthermore, the proportion of participants with poor HbA1c levels was remarkably smaller in BASE-II. (Table 2). Accordingly, the median HbA1c was significantly lower in the BASE-II compared to the BASE sample (5.7% vs. 5.9%, p = 0.002).

Prevalence of antidiabetic drug use was similar, as was the prevalence of self-reported diabetes mellitus (Table 1).

### Cohort differences in self-rated health

Self-rated health (SRH) was lower among the later-born than among earlier-born individuals. As shown in Fig 2C, significantly smaller proportions in the BASE-II than in BASE sample rated their current health as *very good* or *good*, *while* most BASE-II participants (79.9%) rated their health as satisfactory or fair only. Equal proportions of both samples had poor SRH (11.4% in BASE-II vs. 11.6% in BASE).

### Follow up analyses

Table 3 presents the results from linear regression analyses with the LS7 score as the dependent variable, and cohort membership (BASE vs. BASE-II), age, sex, education, and cardiovascular disease as independent variables. Of the correlates tested and when all other variables had been part of the model prediction, only membership in BASE-II (*β* = –0.335, *p* <0.001) was significantly associated with a higher LS7 score, denoting better cardiovascular health. Notably, a history of cardiovascular outcomes did not show significant association with the LS7 score. Likewise, there was no indication of additional interaction effects involving cohort.

**Table 3:** Standardized prediction effects (β) from regression analyses of Life’s simple 7 score.

	β	*p*
Age	0.066	0.234
Sex (1 = male, 2 = female)	-0.053	0.391
education (centered at cohort mean)	0.004	0.99
Cohort (0 = BASE-II, 1 = BASE)	-0.335	<0.001
CV disease (0 = no, 1 = yes)	-0.098	0.136
R^2^ = 0.128, F = 6.825		

## Discussion

The emerging picture of trends in modifiable indicators of cardiovascular health, comparing serial cross-sectional data obtained about 20 years apart in 1990–1993 (BASE) and 2009–2014 (BASE-II) was heterogeneous. Overall, we found that the burden of cardiovascular risk factors had decreased. The mean LS7 score was significantly higher in the sample derived from the BASE-II cohort, indicating better CV health in the later-born individuals. In detail, 17.4% vs. 6.6% (BASE-II vs. BASE) presented optimal CVH, whereas only 3.3% had inadequate CVH in BASE-II compared to 19.8% in BASE.

Overall, this is encouraging and the suggested improvements in diet, smoking, physical activity, diabetes and cholesterol are very welcome. On the other hand, there were also unfavorable aspects:

First, there was no indication of improvements in blood pressure (BP) in later-born individuals as compared to earlier-born individuals. Although more people were treated with modern antihypertensive drugs, the mean BP even resulted to be higher in the later-born BASE-II sample. Consistent with this finding, some previous studies also found that among older adults (aged 60 years and older) hypertension had increased [22], comparing prevalent cases across two periods (1988–1994 and 1999–2004), or was unchanged [23], whereas the general trend in Germany and central Europe is towards decreasing blood pressure [24, 25].

Population-based studies have suggested that up to 75% of hypertension is attributable to overweight and obesity [26]. Accordingly, in the present study, a lack of improvement in hypertension was paralleled by largely unchanged BMI. In fact, recent data from Europe and the US suggested a slowing or leveling of the prior secular trend of rising prevalence of overweight and obesity [12, 24, 27, 28]. Admittedly, post-hoc power analyses indicated that statistical power of the present study to detect differences in BMI-categories was low, and additional consideration of waist circumference (WC), which was significantly higher in the later-born BASE-II sample, suggested that we might have missed existing BMI differences. Indeed, the increase in WC may explain in part the lack of decline in BP.

In contrast to blood pressure and BMI trends, the prevalence of high total cholesterol (≥240 mg/dL) was significantly lower in the later-born individuals. Consistent with our findings, surveys from Europe and the US showed similar secular downward trends in total cholesterol and/or LDL-C over the past 20 years [24, 29]. This trend was in part attributed to the increasing number of individuals using lipid-lowering medications among the older age group. Our data reflect this with twice the number of subjects treated with lipid-lowering medications in the later born sample (17.4% in BASE-II vs. 9.1% in BASE). Admittedly, the trend of falling TC has begun even before the era of statins, and substantial evidence has identified diet as the major contributor to this trend [30].

Whereas nationally representative data from Germany have shown an unchanged prevalence of type II diabetes during the past two decades [31], US and European studies have consistently shown increases in diabetes across all ages [11], particularly among those aged 60 years and older [32, 33].

Comparison of matched samples from BASE-II and BASE demonstrated a favorable trend for HbA1c. The proportion of individuals with optimal HbA1c of <5.7% was significantly greater in the later-born sample (46.2% vs. 35.5%, p = 0.001) and at the same time fewer participants showed a poor HbA1c ≥6.5% (8.4% vs. 30.6%, p = 0.001). The prevalence of antidiabetic medication use did not differ between BASE and BASE-II, which may indicate that the better HbA1c could be rather attributed to favorable changes in health factors and behaviors (e.g. diet and lifestyle) than to improvements in medical treatment.

Given the unimproved high rates of overweight and obesity in the later-born individuals (66.7%), there is an apparent contradiction, as one would have expected a similar or concordant trend for diabetes, too [34]. However, based on representative German data, Finger et al. also documented decreasing serum-glucose levels for the general population, while the prevalence of obesity increased [24], which is consistent with our results. Supposedly, a leveling of the BMI-trend, as suggested by our data, in conjunction with beneficial changes in other indicators of cardiovascular health are probably sufficient to explain this apparent “decoupling” of adiposity and diabetes. Such an interpretation would be consistent with empirical evidence suggesting a decoupling of BMI with blood pressure and cholesterol [34].

The significance of obesity as risk factor for insulin resistance is well established. However, at the same time, there is substantial evidence that a healthy diet (particularly a good macronutrient composition) and regular exercise can effectively lower CVD and diabetes risk. [35, 36]—even in overweight and obese individuals.

Indeed, whereas in the BASE sample 50% of participants reported to be physically inactive, in the BASE-II sample only 26.8% were inactive and the rest exercised on a very regular basis. This trend is in line with findings of Krug et al. showing that the prevalence of *lack of exercise* dropped between 1990–1992 and 2008–2011 in older German adults [37]. Moreover, our data likewise suggest that among later-born older adults, a significantly greater proportion adheres to a healthy diet, whereas among the earlier-born BASE participants still a large proportion had a rather poor diet (42%). Other data from Germany also reflect improvements in dietary habits among older adults [38].

At last, fortunately, among the later-born individuals significantly fewer people were current smokers (4% vs. 19%, p < 0.001), which is in line with German representative data [39], and which is, at least in part, attributable to public policies, like smoke-free laws and an increased awareness of the smoking-associated health risks.

As another interesting aspect, examining cohort differences over 20 years against the background of relevant innovations in healthcare and health behavior, we considered self-rated health (SRH). Interestingly, the proportion of individuals rating their current health as only satisfactory or fair was significantly higher in the later-born sample. In contrast, we have seen that objective cardiovascular health was significantly better in later-born individuals. Thus, one may conclude that BASE-II participants had a more critical appraisal of their own health associated with higher expectations. In another study, examining baby boomers and preboomers, the authors also found lower SRH for the later born cohort [40], and they concluded that the later-born cohort may have adopted ideas of preventive medicine and health promotion and therefore may use these ideas when assessing their own health, which is also plausible in the context of the present analysis. A higher awareness of the link between a life style and CVH may be a main driving force accounting for the improved health behavior, and improved CVH as observed in our study. In this respect, recent findings [41], which were also based on cohort comparisons of BASE and BASE-II are interesting to consider. Here we could show better well-being and lower levels of external control beliefs among later-born individuals compared to earlier-born individuals.

### Limitations

There are some limitations of this study. First, propensity score matching procedures were successful in making the examined BASE and BASE-II samples comparable regarding socio-demographic characteristics. However, since the two matched samples were not random samples of the original cohorts, naturally we cannot preclude any selection bias as a consequence of propensity-score matching (PSM). Thus, the two matched samples cannot be regarded as representative of the original cohorts. Primarily, the original cohorts had a very different age-distribution, and they reasonably differed in terms of education, which is shown in Fig 1 and in the description of the cohorts in the methods section. Moreover, BASE was a population-based sample, whereas BASE-II was a convenience sample. However, the response rate in BASE was rather low (27%), and participants were proven positively selected regarding education, health, and cognition [15], which was also shown for BASE-II [14]. As detailed earlier [18], the amount of sample selectivity was in essence comparable between BASE and BASE-II. We used propensity-score matching to control for differences in sampling strategies between BASE and BASE-II and relevant individual characteristics, which made the two subsamples analyzed here reasonably comparable.

In the matched BASE sample, slightly more participants had a history of stroke and CHD. We met this by conducting follow-up analyses. According to these, history of stroke/CHD did not show significant association with the LS7 score (Table 3).

As a consequence of the PSM procedure our sample size (n = 240) was rather small. Thus, although we saw relevant differences in proportions, sometimes statistical significance was not reached (e.g. BMI-categories). According to post-hoc power analysis, with the given sample size we cannot rule out that existing differences in BMI categories were missed. Furthermore, self-reported physical activity, diet and smoking were subject to recall and desirability biases. Finally, the serial cross-sectional study design does not permit causal interference.

## Conclusions

To conclude, this study suggests that CVH of older German adults has significantly improved over time from 1990–93 to 2009–2014, yet the emerging picture is not unanimously positive and there is still need for improvements. Overall, the depicted trends in central indicators of cardiovascular health among community-dwelling older adults are consistent with the reported downward trends in mortality due to cardiovascular risk factors and heart disease in Germany since 1990. Moreover, our data suggest that later-born cohorts of older adults may have higher expectations about their health and indeed adopted ideas of preventive medicine and health promotion.
